# Independence of the mortality of severely injured patients from types of transport and hospital level in a well-developed trauma network

**DOI:** 10.1007/s00068-025-02997-2

**Published:** 2025-10-28

**Authors:** Antonio Ernstberger, Daniel Popp, Jakob Holtfrerich, Florian Baumann, Volker Alt, Claudius Thiedemann

**Affiliations:** Department for Trauma Surgery, University Hospital Regensburg, Franz-Josef-Strauss-Allee 11, Regensburg, 93053 Germany

**Keywords:** Multiple trauma, Polytrauma, Trauma network, Mode of transport, HEMS, GEMS

## Abstract

**Introduction:**

Despite over 50 years of research, trauma remains a significant global health issue. In addition to medical advancements, establishing trauma networks appears to positively impact the survival rates of severely injured patients. The influence of the type of transport (helicopter emergency medical service [HEMS] vs. ground emergency medical service [GEMS]) on mortality depending on the destination hospital in an established trauma network is currently unclear. The objective of the study was to evaluate this line of questioning within the context of an entire trauma network.

**Materials and methods:**

Data from all trauma room patients in the first established trauma network in Germany over a period of 24 months were analyzed. Although the data was collected prospectively and entered the TraumaRegister DGU^®^ database, it was analyzed retrospectively in relation to the research question. The trauma network served a population of approximately 2.3 million people in an area of about 20,000 square kilometers in a predominantly rural area, which comprised 2 Level I, 8 Level II, and 15 Level III hospitals. A 24/7 dual-use helicopter and three other rescue helicopters were available during the day. Two additional rescue helicopters from other networks were potentially available during the night. Patients with an Injury Severity Score (ISS) ≥ 16 were included in this study. Patients with secondary admission, those transferred or discharged within 48 h, and cases with missing Revised Injury Severity Classification II (RISC II) were excluded. Groups were divided according to target hospital level (I, II, or III) and transport type (HEMS or GEMS). A total of 5 groups (LI-H, LI-G, LII-H, LII-G, LIII-G) were available for evaluation, as no patient was transported by helicopter to a Level III hospital during the study period. Univariate statistics were performed using the Chi-Square and Kruskal-Wallis tests. The significance level was set at *p* < 0.05. Post-hoc analyses were performed for significant results. In addition, multivariate analyses were carried out to identify masked correlations. RISC II was taken to calculate the Standardized Mortality Ratio (SMR).

**Results:**

After applying the inclusion and exclusion criteria, 887 of the 2,596 available cases were included in the study. The distribution to the study groups was as follows: LI-H 20.6% (*n* = 183), LI-G 17.0% (*n* = 151), LII-H 21.6% (*n* = 192), LII-G 35.9% (*n* = 318), LIII-G 4.8% (*n* = 43). The univariate analysis of patient characteristics revealed significant differences in seven out of eight variables (Age, Sex, Classification of American Society of Anesthesiologists (ASA), Abbreviated Injury Scale (AIS) of the region head, ISS, New Injury Severity Score (NISS), RISC II). Patients transported to Level I hospitals by HEMS (LI-H group) tended to be younger, have a lower ASA score, and sustain more severe injuries with a high risk of death (RISC II). The evaluation of the preclinical and clinical courses continued to demonstrate the heterogeneity of the patient population, which was characterized by worse vital signs, an increased need for infusion and transfusion, higher intubation rates, longer ventilation times, and longer lengths of stay in ICUs and overall for patients in Level I hospitals, particularly for HEMS transports. Despite these differences, the outcome was comparable. There were no significant differences in mortality between the groups, either unadjusted (LI-H 19,1% (*n* = 35), LI-G 24,5% (*n* = 37), LII-H 20,3% (*n* = 39), LII-G 17,0% (*n* = 54), LIII-G 14,0% (*n* = 6), *p* = 0,326) nor adjusted. Similarly, the multivariate analysis did not reveal any correlations between mortality, hospital level and mode of transportation.

**Discussion:**

While a positive influence on mortality through the formation of trauma networks can be found in literature, the results for the type of transportation are inconsistent. However, there are some indications that HEMS transports may offer a survival advantage. These papers often show rather homogeneous patient characteristics, which were not found in this study. This study demonstrated differentiated prehospital patient selection, in which patients were transported to the appropriate hospital via the most suitable means within a trauma network, ensuring a comparable outcome for all patients. In the future, further optimization and simplification of preclinical patient selection could be achieved by transmitting accident data stored in modern cars that is currently unavailable to emergency services and hospitals.

## Introduction

Trauma is still a global burden of disease after more than 50 years of research [1–3]. Road traffic accidents (RTA) are the number one cause of death for 10–49 year olds worldwide and rose from 8th to 7th place among the leading causes of death between 1990 and 2019 [1].

In contrast, mortality after RTA has been significantly reduced in industrialized countries in recent decades. Whereas Germany recorded around 20,000 RTA deaths in the 1970s, this figure was reduced to under 3,000 in the 2020s [4]. The reduction in mortality after RTA was accompanied by an increase in the probability of survival after multiple trauma in general [5].

Many initiatives and tools have been used to minimize mortality from injuries. The formation of trauma networks appears to be a useful way of further reducing mortality and was successfully implemented in the USA in the 1980s [6, 7]. A growing number of trauma networks have been set up in various countries [8–12]. In Germany, the philosophy of trauma networks was first published in 2006 with the White Paper on the care of severely injured patients by the German Society for Trauma Surgery (DGU) and has been continuously developed since then [13–15].

The White Paper describes the structural requirements for a hospital wishing to participate in the care of seriously injured patients, graded according to care levels, and obliges the hospitals in a region to form a trauma network. The aim is to improve the quality of care not just for one hospital, but for an entire region [15, 16]. It has been determined that a patient must be admitted to a Level I (Germany ÜTZ) or Level II (Germany RTZ) center within 30 min. If transportation time is too long or the patient is too unstable, they must be taken to a Level III center for stabilization and transferred from there. This philosophy aims to offer every traumatized person the same chances of survival, regardless of where they have been injured. A condition that demonstrably did not exist in Germany in 2006 [17, 18]. This improvement in the quality of results should be achieved through a specified structural quality, an improvement in process quality through, among other things, the mandatory Advanced Trauma Life Support (ATLS) training of physicians and the networking of clinics to form a trauma network [15, 16]. This trauma network has been implemented throughout Germany since 2015. The structural quality has demonstrably increased in the hospitals, and a harmonization of the quality of results within a network has also been demonstrated [19–21].

The impact of the type of transportation used in combination with the destination hospital on the quality of the outcome is not yet fully understood. Several studies have shown that before the implementation of trauma networks in Germany, there was a difference in the mortality of multiple injured patients depending on the type of transport [22–24]. There is also some indication in the international literature of a survival advantage for patients with Helicopter Emergency Medical Services (HEMS) transport [25, 26].

The aim of this study was to evaluate the mortality of multiple injured patients depending on the type of transport and the level of the destination hospital in a developed trauma network.

The hypothesis, based on the objectives of the White Paper on the care of severely injured, was that no difference in mortality would be observed - the right patient would be admitted to the right hospital at the right time with the right transport system.

## Materials and methods

The research is a multicenter study of the hospitals of a defined trauma network with prospective data collection. The trauma network comprised two Level I centers (German ÜTZ), 8 Level II centers (German RTZ) and 15 Level III centers (German LTZ) covering an area of 20,000 square kilometers with a total population of 2.3 million. The network was the first to be certified in Germany and was well developed and trained at the time of data collection. Three HEMS were stationed in the study region (available sunrise to sunset) and one 24/7 dual-use helicopter (rescue and intensive care transfer). At night, the area was covered by this helicopter and 2 others 24/7 from neighboring trauma networks. In Germany, the response time for Ground Emergency Medical Service (GEMS) is 15 min, and not only are the HEMS manned by emergency physicians, but the GEMS are also supported by physicians who are always on site for the multiple trauma patients [21, 27].

The data of all severely injured patients who were admitted to a trauma room in a hospital in the network were prospectively recorded over a period of 24 months from March 2012 to February 2014 and transferred to the ‘TraumaRegister^QM^ DGU^®^’ database [3]. The database has existed since 1993, is anonymized and Utstein-compliant and is constantly updated [28, 29]. In addition to demographics and the circumstances of the accident, the data set includes clinical findings and treatment at the scene, in the trauma room, and the course of events including surgery, ICU stay and complications up to discharge or death. Several scoring systems were used in the data set, including GCS (Glasgow Coma Score [30]), AIS (Abbreviated Injury Scale [31]), ISS (Injury Severity Score [32]), NISS (New Injury Severity Score [33]), ASA (American Society of Anesthesiologists physical status classification system [34]), GOS (Glasgow Outcome Scale [35]) and RISC II (Revised Injury Severity Classification II [36]).

The SMR (Standardized Mortality Ratio) was calculated as the percentage of deaths divided by the expected mortality of the RISC II. An SMR < 1 indicates an increased probability of survival compared with the expected probability, and an SMR > 1 indicates a decreased probability.

Cases with an ISS ≥ 16 were included in the study dataset, regardless of age and level of the destination hospital. Patients who were transferred or discharged within 48 h and cases with missing RISC II data were excluded.

The study sample was subdivided according to hospital care levels (I/II/III) and type of transport (HEMS/GEMS). There were 5 groups LI-H/LI-G, LII-H/LII-G and LIII-G, as no patients were admitted to Level III hospitals by HEMS. There were also no patients in our study population who were brought to hospital by private transportation.

### Statistical analysis

The 5 groups were described as a function of the variables (dichotomous/metric/ordinal) with percentages or means/standard deviations. The univariate analysis was carried out using the Chi-Square test (dichotomous or ordinal variables) and the Kruskal-Wallis test (metric variables). The significance level was set at 0.05. If there was a significant difference between all groups in the univariate analysis, a post-hoc test was performed. The post-hoc tests considered Bonferroni correction. The mortality was adjusted via the SMR and tested for significance using t-test. Subsequently, multivariate regression analyses were conducted to clarify whether the results from the univariate analysis hold or whether independent predictors are unmasked. In particular, to test whether there was an effect due to hospital level and/or type of transport.

The study was authorized by the Ethics Committee of the University of Regensburg (number 10–101-0077), funded by the German Federal Ministry of Education and Research (number 01GY1153) and registered with the German Network for Health Research (VfD_Polyqualy_12_001978) and the German Clinical Trials Register (number DRKS00010039).

## Results

### Study population

**Fig. 1:**
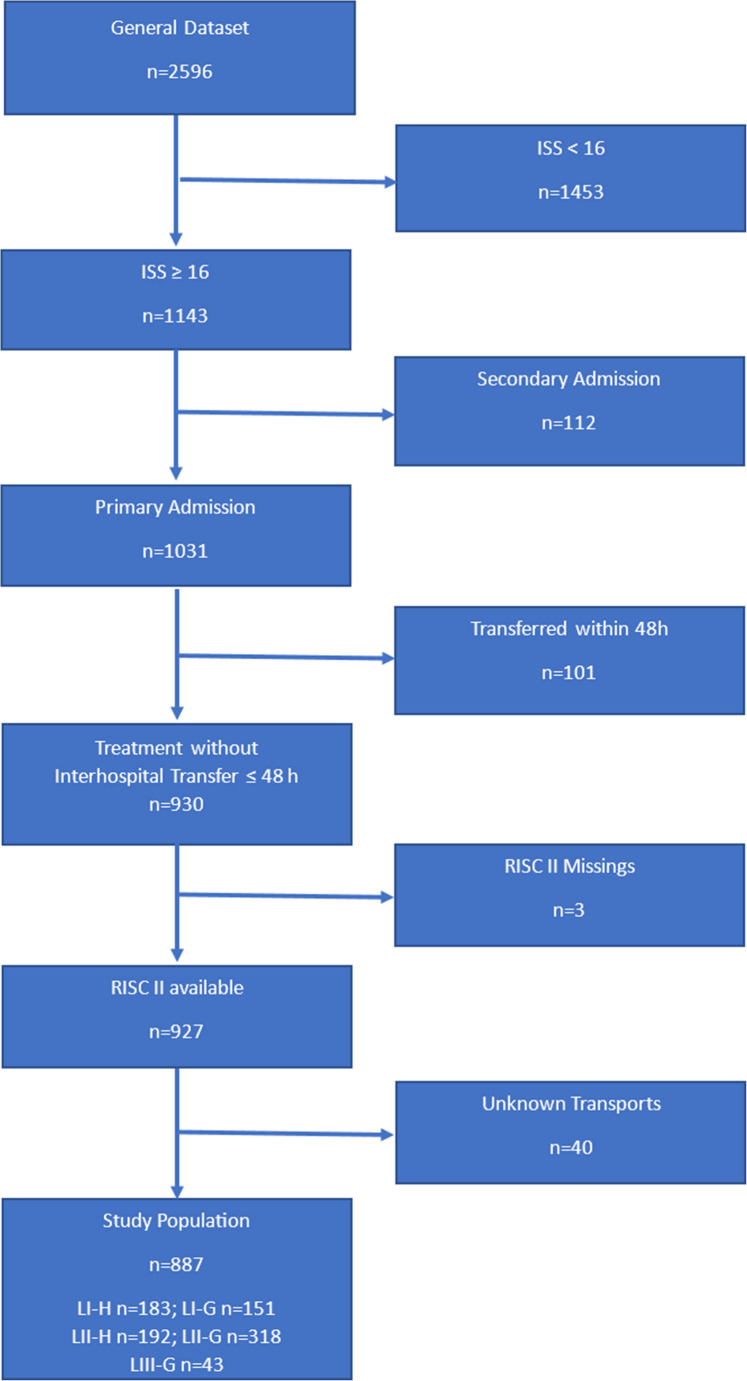
Study population

The primary data set contained a total of 2596 cases. After applying the inclusion and exclusion criteria, 887 cases were available for the study. 334 patients were admitted to Level I hospitals (37.7% of the study population), 510 patients (57.5%) to Level II hospitals and 43 patients (4.8%) to Level III hospitals. A total of 375 patients (42.3% of the study population), including 54.8% (*n* = 183) of patients at Level I centers and 29.6% (*n* = 151) of patients at Level II centers (*p* < 0,001), were transported by HEMS. No patients were admitted to Level III centers by HEMS. For the entire study sample, the distribution was as follows: LI-H 20.6% (*n* = 183), LI-G 17.0% (*n* = 151), LII-H 21.6% (*n* = 192), LII-G 35.9% (*n* = 318), LIII-G 4.8% (*n* = 43) (Fig. 1).

### Patient characteristics

The data showed significant differences for the study groups. The values for age, percentage of males and ASA differed significantly. For these variables, the groups transported by HEMS to Level I or Level II hospitals showed a high degree of similarity. The groups transported by GEMS - regardless of the level of care - were also similar in terms of age and ASA. The gender ratio showed a minimum in the LI-G group (64.2% males) and a maximum in the LIII-G group (86.0% males).

The overall injury severity (ISS and NISS) and the calculated probability of death (RISC II) also differed significantly between the groups. However, the groups at hospital level were now somewhat more similar regardless of the type of transport, and the groups of Level I hospitals were significantly different from the other hospital levels in the post-hoc analysis. Overall injury severity and RISC II decreased steadily with hospital level and transport type, except for RISC II for LI-H and LI-G. More severely injured patients with a higher probability of death were more frequently transferred to Level I hospitals. The results for severe traumatic brain injury (TBI) also showed the difference between levels, but the proportion was higher in the GEMS-transported groups than in the HEMS groups. The high percentage of severe TBIs in the Level I GEMS group, which represented the maximum value, was noticeable (Table 1).

**Table 1:** Patient characteristics

	LI-H *n* = 183	LI-G *n* = 151	LII-H *n* = 192	LII-G *n* = 318	LIII-G *n* = 43	*p*
Age (Mean/SD)	42,4/±22,1	51,8/±22,7	47,8/±20,4	51,3/±21,6	55,1/±23,4	< 0,001
post-hoc	LI-H/LI-G *p* = 0,001, LI-H/LII-G *p* < 0,001, LI-H/LIII-G *p* = 0,006					
Sex Male (%/n)	77,6% (142)	64,2% (97)	74,0% (142)	73,3% (233)	86,0% (37)	0,020
post-hoc	LI-H/LI-G *p* = 0,007, LI-G/LIII-G *p* = 0,006					
ASA (Mean/SD)	1,4/±0,6	1,7/±0,8	1,4/±0,7	1,6/±0,7	1,9/±0,8	< 0,001
*Missing (%/n)*	*1*,*6% (3)*	*3*,*3% (5)*	*7*,*3% (14)*	*8*,*8% (28)*	*14*,*0% (6)*	
post-hoc	LI-H/LI-G *p* < 0,001, LI-H/LII-G *p* = 0,025, LI-H/LIII-G *p* < 0,001, LI-G/LII-H *p* = 0,004, LII-H/LIII-G *p* = 0,003					
Penetrating Injury (%/n)	4,4% (8)	4,0% (6)	3,2% (6)	2,3% (7)	0% (0)	0,505
*Missing (%/n)*	*0*,*5% (1)*	*0*,*7% (1)*	*2*,*1% (4)*	*4*,*4% (14)*	*7*,*0% (3)*	
AIS Head ≥ 4 (%/n)	36,6% (67)	49,7% (75)	25,5% (49)	29,2% (93)	23,3% (10)	< 0,001
post-hoc	LI-G/LI-H *p* < 0,001, LI-G/LII-G *p* < 0,001, LI-G/LIII-G *p* < 0,001					
ISS (Mean/SD)	32,3/±15,1	29,4/±13,7	26,8/±11,3	24,2/±9,6	22,8/±8,3	< 0,001
post-hoc	LI-H/LII-H *p* = 0,002, LI-H/LII-G *p* < 0,001, LI-H/LIII-G *p* < 0,001, LI-G/LII-G *p* < 0,001, LI-G/LIII-G *p* = 0,033					
NISS (Mean/SD)	39,3/±17,1	36,0/±16,0	32,6/±13,3	29,7/±11,5	29,0/±12,0	< 0,001
post-hoc	LI-H/LII-H *p* = 0,005, LI-H/LII-G *p* < 0,001, LI-H/LIII-G *p* < 0,001, LI-G/LII-G *p* = 0,001, LI-G/LIII-G *p* = 0,049					
RISC II (Mean/SD)	21,7/±31,7	24,3/±31,6	15,3/±25,2	14,9/±26,0	13,1/±25,7	0,001
post-hoc	LI-G/LII-G *p* < 0,001					

Percentages refer respectively to groups LI-H, LI-G, LII-H, LII-G, LIII-G. *ASA* Classification of American Society of Anesthesiologists, *AIS* Abbreviated Injury Scale, *ISS* Injury Severity Score, *NISS* New Injury Severity Score, *RISC II* Revised Injury Severity Classification II. If no missing line was specified, the completeness was 100%

### Preclinical evaluation

Preclinical data for resuscitation and crystalloid infusion variables did not differ between study groups. All other prehospital characteristics (GCS, blood pressure, intubation, infusion of colloidal fluids, prehospital duration) were significantly different. The initial GCS was, analogous to the ISS/NISS and RISC II, continuously ascending for the hospital levels and transport types. In contrast, prehospital intubation and colloid infusion were more common in the HEMS groups.

It should be noted that the use of colloidal infusions has declined significantly in recent years. In cases of massive blood loss, colloid infusions are still indicated, meaning that the significant result of the evaluation is valid [37]. 

The LI-H group included the patients with lowest blood pressure and the longest preclinical time. For Level I and Level II hospitals, the type of transport seems to be decisive for intubation and infusion regimes (Table 2).

**Table 2:** On scene values

	LI-H *n* = 183	LI-G *n* = 151	LII-H *n* = 192	LII-G *n* = 318	LIII-G *n* = 43	*p*
GCS on scene (Mean/SD)	10,2/±4,8	10,4/±4,9	10.9/±5,0	12,3/±4,2	13,6/±3,2	< 0,001
*Missing (%/n)*	*1*,*6% (3)*	*2*,*6% (4)*	*1*,*6% (3)*	*5*,*7% (18)*	*4*,*7% (2)*	
post-hoc	LI-H/LII-G *p* < 0,001, LI-H/LIII-G *p* < 0,001, LI-G/LII-G *p* < 0,001, LI-G/LIII-G *p* < 0,001, LII-H/LII-G *p* = 0,037, LII-H/LIII-G *p* = 0,027					
BP sys. on scene (mmHg) (Mean/SD)	113,0/±41,9	124,0/±41,8	123,6/±32,6	125,5/±34,9	127,0/±29,8	0,014
*Missing (%/n)*	*5*,*5% (10)*	*13*,*9% (21)*	*10*,*4% (20)*	*7*,*5% (24)*	*7*,*0% (24)*	
post-hoc	LI-H/LII-G *p* = 0,006					
Intubation on scene (%/n)	68,9% (126)	43,9% (65)	50,5% (97)	24,5% (77)	14,0% (6)	< 0,001
*Missing (%/n)*	*0% (0)*	*2*,*0% (3)*	*0% (0)*	*1*,*3% (4)*	*0% (0)*	
post-hoc	LI-H/LI-G *p* < 0,001, LI-H/LII-H *p* < 0,001, LI-H/LII-G *p* < 0,001, LI-H/LIII-G *p* < 0,001, LI-G/LII-G *p* < 0,001, LI-G/LIII-G *p* < 0,001, LII-H/LII-G *p* < 0,001, LII-H/LIII-G *p* < 0,001					
Reanimation on scene (%/n)	6,0% (11)	6,7% (10)	3,6% (7)	5,2% (16)	4,8% (2)	0,763
*Missing (%/n)*	*0*,*5% (1)*	*0*,*7% (1)*	*0% (0)*	*2*,*5% (8)*	*2*,*3% (1)*	
Crystalloid Infusion (ml) (Mean/SD)	839,1/±501,6	840,0/±716,6	872,4/±559,1	743,3/±458,7	651,4/±361,0	0,070
Colloid Infusion (ml) (Mean/SD)	288,3/±385,5	224,7/±406,8	254,0/±342,0	143,1/±283,8	157,9/±291,2	< 0,001
post-hoc	LI-H/LI-G *p* < 0,001, LII-H/LII-G *p* = 0,007					
Time to ER (min) (Mean/SD)	64,9/±22,8	45,0/± 16,6	45,4/±23,2	38,5/±19,4	38,4/±16,8	< 0,001
*Missing (%/n)*	*45*,*9% (84)*	*35*,*1% (53)*	*49*,*0% (94)*	*39*,*9% (127)*	*11*,*6% (5)*	
post-hoc	LI-H/LI-G *p* < 0,001, LI-H/LII-H *p* < 0,001, LI-H/LII-G *p* < 0,001, LI-H/LIII-G *p* < 0,001, LI-G/LII-G *p* = 0,016, LII-H/LII-G *p* = 0,032					

Percentages refer respectively to groups LI-H, LI-G, LII-H, LII-G, LIII-G. *Time to ER* Time between arrival of emergency physician on scene to arrival hospital. If no missing line was specified, the completeness was 100%

### Trauma room results

Except for the mean base excess (BE) and the percentage of patients who received a CT scan (cranial computed tomography (CCT) or whole body computed tomography (WBCT)), all variables recorded in the trauma room were also significantly different between the study groups. The LI-H group again showed a minimum in blood pressure, Quick and hemoglobin value (Hb). The Hb of 12.1 g/dl was similarly measured in the LII-H group. Nevertheless, the mean value of transfused packed red blood cells (PRBC) decreased continuously between the LI-H/LI-G/LII-H/LII-G groups. More PRBCs were transfused in Level III than in Level II hospitals (0,7 vs. 0,5). The percentage of patients transfused behaved similarly. The LI-H and LI-G groups had the most frequent mass transfusions, in Level III hospitals, no patient underwent mass transfusion during the observation period. Group LIII-G showed a maximum time to CT of 45.4 min, which was twice as long as in the other groups. Across all groups and all variables, the hospital levels separated better than the means of transportation (Table 3).

**Table 3:** Trauma room values

	LI-H *n* = 183	LI-G *n* = 151	LII-H *n* = 192	LII-G *n* = 318	LIII-G *n* = 43	*p*
BP sys. (mmHg) (Mean/SD)	116,3/±32,8	126,1/±38,2	125,3/±31,9	124,9/±34,6	129,7/±30,0	0,008
*Missing (%/n)*	*7*,*1% (13)*	*21*,*9% (33)*	*9*,*4% (18)*	*6*,*3% (20)*	*9*,*3% (4)*	
post-hoc	LI-H/LII-G *p* = 0,009					
Base Excess (mmol/l) (Mean/SD)	−4,0/±6,0	−3,9/±5,9	−2,7/±4,2	−2,3/±5,1	−2,3/±2,6	0,082
*Missing (%/n)*	*7*,*1% (13)*	*25*,*8% (39)*	*19*,*3% (37)*	*26*,*1% (83)*	*27*,*9% (12)*	
Quick (Mean/SD)	71,9/±22,6	78,0/±24,2	76,8/±20,3	83,1/±21,5	86,6/±21,1	< 0,001
*Missing (%/n)*	*2*,*7% (5)*	*6*,*0% (9)*	*5*,*7% (11)*	*6*,*6% (21)*	*2*,*3% (1)*	
post-hoc	LI-H/LI-G *p* = 0,011, LI-H/LII-G *p* < 0,001, LI-H/LIII-G *p* < 0,001, LII-H/LII-G *p* < 0,001, LII-H/LIII-G *p* = 0,035					
First Hb value (g/dl) (Mean/SD)	12,1/±3,0	12,4/±2,9	12,1/±2,8	12,9/±2,5	13,2/±2,6	0,003
*Missing (%/n)*	*0*,*5% (1)*	*2*,*6% (4)*	*2*,*1% (4)*	*4*,*4% (14)*	*2*,*3% (1)*	
post-hoc	LI-H/LII-G *p* = 0,044, LII-H/LII-G *p* = 0,024					
Transfusion of PRBC (Mean/SD)	2,3/±7,0	0,8/±3,0	0,5/±2,8	0,5/±4,1	0,7/±1,9	< 0,001
post-hoc	LI-H/LII-H *p* < 0,001, LI-H/LII-G *p* < 0,001					
Patients transfused (%/n)	21,3% (39)	12,6% (19)	6,8% (13)	6,0% (19)	14,0% (6)	< 0,001
post-hoc	LI-H/LII-H *p* < 0,001, LI-H/LII-G *p* < 0,001					
Mass transfusion (%/n)	7,1% (13)	2,6% (4)	0,5% (1)	1,3% (4)	0% (0)	< 0,001
post-hoc	LI-H/LII-H *p* < 0,001, LI-H/LII-G *p* < 0,001					
Transfusion of FFP (Mean/SD)	2,6/±7,0	0,8/±3,7	0,2/±1,4	0,4/±3,8	0,1/±0,4	< 0,001
post-hoc	LI-H/LI-G *p* < 0,001, LI-H/LII-H *p* < 0,001, LI-H/LII-G *p* < 0,001, LI-H/LIII-G *p* = 0,004					
Patients with FFP transfusion (%/n)	20,2% (37)	7,3% (11)	3,6% (7)	3,5% (11)	4,7% (2)	< 0,001
post-hoc	LI-H/LI-G *p* < 0,001, LI-H/LII-H *p* < 0,001, LI-H/LII-G *p* < 0,001					
CT-Scan (WB or CCT) (%/n)	95,6% (174)	94,7% (143)	97,4% (184)	93,9% (292)	90,0% (36)	0,263
*Missing (%/n)*	*0*,*5% (1)*	*0% (0)*	*1*,*5% (3)*	*2*,*2% (7)*	*7*,*0% (3)*	
Time door to CT (min) (Mean/SD)	21,8/±7,7	22,7/±18,1	18,3/±8,8	24,3/±20,8	45,4/±39,5	< 0,001
*Missing (%/n)*	*8*,*2% (15)*	*15*,*9% (24)*	*5*,*7% (11)*	*19*,*2% (61)*	*39*,*5% (17)*	
post-hoc	LI-H/LII-H *p* < 0,001, LI-H/LIII-G *p* = 0,005, LI-G/LII-H *p* = 0,032, LI-G/LIII-G *p* = 0,001, LII-H/LII-G *p* = 0,025, LII-H/LIII-G *p* < 0,001, LII-G/LIII-G *p* < 0,001,					

Percentages refer respectively to groups LI-H, LI-G, LII-H, LII-G, LIII-G. *BP* Blood Pressure, *Hb* Hemoglobin, *PRBC* Packed Red Blood Cells, Mass transfusion: more than 10 PRBC, *FFP* Fresh Frozen Plasma, *CT* Computed Tomography, *WB* Whole Body, *CCT* Cranial Computed Tomography. If no missing line was specified, the completeness was 100%

### Clinical course and secondary endpoints

As in the previous sections, the differences between the study groups were also significant in the clinical course, except for the variable total length of hospital stay. The LI-H group presented the maximum of required ICU therapy (97.8%) and ventilation therapy (77.9%), duration of ventilation (5.7 d) and length of ICU stay (10.5 d). Again, there was a continuous decrease in these variables across the study groups, with exception of percentage of ICU therapy. While the percentage of patients receiving ICU therapy in Level II hospitals was below 90%, Level I and Level III hospitals had a rate of > 90%. Once more, the hospital level groups appeared more similar than the transportation groups (Table 4).

**Table 4:** Clinical course/secondary endpoints

	LI-H *n* = 183	LI-G *n* = 151	LII-H *n* = 192	LII-G *n* = 318	LIII-G *n* = 43	*p*
ICU therapy (%/n)	97,8% (179)	92,1% (139)	87,0% (167)	89,6% (285)	93,0% (40)	0,003
post-hoc	LI-H/LII-H *p* < 0,001, LI-H/LII-G *p* < 0,001					
Stay on ICU (d) (Mean/SD)	10,5/±11,0	9,5/±10,0	7,8/±10,0	7,1/±10,2	4,7/±5,9	< 0,001
post-hoc	LI-H/LII-H *p* = 0,006, LI-H/LII-G *p* < 0,001, LI-H/LIII-G *p* = 0,004, LI-G/LII-G *p* = 0,038					
Ventilation therapy (%/n)	77,9% (141)	62,3% (94)	58,3% (112)	42,5% (133)	31,7% (13)	< 0,001
*Missing (%/n)*	*1*,*1% (2)*	*0% (0)*	*0% (0)*	*1*,*6% (5)*	*4*,*7% (2)*	
post-hoc	LI-H/LI-G *p* = 0,002, LI-H/LII-H *p* < 0,001, LI-H/LII-G *p* < 0,001, LI-H/LIII-G *p* < 0,001, LI-G/LII-G *p* < 0,001, LI-G/LIII-G *p* < 0,001, LII-H/LII-G *p* < 0,001, LII-H/LIII-G *p* = 0,002					
Ventilator days (Mean/SD)	5,7/±8,1	4,5/±7,1	4,4/±7,7	3,2/±7,5	1,0/±2,4	< 0,001
*Missing (%/n)*	*1*,*1% (2)*	*0% (0)*	*1*,*0% (2)*	*1*,*9% (6)*	*4*,*7% (2)*	
post-hoc	LI-H/LII-H *p* = 0,004, LI-H/LII-G *p* < 0,001, LI-H/LIII-G *p* < 0,001, LI-G/LII-G *p* = 0,002, LI-G/LIII-G *p* = 0,002, LII-H/LII-G *p* = 0,017, LII-H/LIII-G *p* = 0,006					
Stay in hospital (d) (Mean/SD)	18,0/±15,6	15,7/±14,5	15,8/±14,5	15,8/±14,6	13,1/±10,5	0,158

Percentages refer respectively to groups LI-H, LI-G, LII-H, LII-G, LIII-G. If no missing line was specified, the completeness was 100%. *ICU* Intensive Care Unit.

### Mortality and SMR

In contrast to previous results, neither absolute mortality nor adjusted mortality showed significant differences. The lowest mortality was found in group LIII-G (14.0%), followed by LII-G (17.0%) and LI-H (19.1%). The lowest SMR was found in the LI-H group (0.88, SD 0.62–1.14), the highest in the LII-H group (1.33, SD 0.95–1.70). Table 5 shows all the results of the post-hoc analyses that were not significant for this outcome. The GOS was also not significantly different (Table 5).

**Table 5:** Outcome/Primary endpoint

	LI-H *n* = 183	LI-G *n* = 151	LII-H *n* = 192	LII-G *n* = 318	LIII-G *n* = 43	*p*
Mortality (%/n)	19,1% (35)	24,5% (37)	20,3% (39)	17,0% (54)	14,0% (6)	0,326
RISC II (Mean/SD)	21,7/±31,7	24,3/±31,6	15,3/±25,2	14,9/±26,0)	13,1/±25,7	0,001
*post-hoc*	*LI-G/LII-G p < 0*,*001*					
SMR (95%-CI)	0,88 (0,62 − 1,14)	1,01 (0,73 − 1,29)	1,33 (0,95 − 1,70)	1,14 (0,87 − 1,42)	1,07 (0,28 − 1,86)	
	LI-H/LI-G *p* = 0,521, LI-H/LII-H *p* = 0,059, LI-H/LII-G *p* = 0,219, LI-H/LIII-G *p* = 0,588, LI-G/LII-H *p* = 0,203, LI-G/LII-G *p* = 0,555, LI-G/LIII-G *p* = 0,869, LII-H/LII-G *p* = 0,437, LII-H/LIII-G *p* = 0,557, LII-G/LIII-G *p* = 0,850					
GOS (Mean/SD)	3,8/±1,6	3,6/±1,7	3,8/±1,5	4,0/±1,5	4,1/±1,3	0,108
*Missing (%/n)*	*0*,*5% (1)*	*0% (0)*	*1*,*0% (2)*	*3*,*1% (10)*	*0% (0)*	

Percentages refer respectively to groups LI-H, LI-G, LII-H, LII-G, LIII-G. *GOS* Glasgow Outcome Scale, *SMR* Standardised mortality rate, *RISC II* Revised Injury Severity Classification II. Completeness of variables was 100%

### Regression analysis

Following the univariate analyses, regression models were created to unmask a previously invisible correlation between mortality, hospital level and/or mode of transport. However, regression analyses confirmed the results of the univariate analyses for the primary endpoint. There was no association between mortality, hospital level, or mode of transport. No influence on mortality could be demonstrated either in the summary of both characteristics or in analyses with separate variables. It was also not possible to demonstrate an influence via the interaction term. The independent variables influencing the mortality of severely injured patients were the severe TBI AIS ≥ 4 (*p* = 0.033), the RISC II (*p* < 0.001), the ASA classification (*p* = 0.001) and the initial blood pressure on scene (*p* = 0.013).

### Number of significant differences from post-hoc analyses

As an addition the number of significant differences from post-hoc analyses was determined. There were 47 significant differences between Level I and Level II hospitals, 41 between the HEMS and GEMS groups. LI-H differed 59 times, LII-H 32 times significantly with other groups (Table 6).

**Table 6:** Number of significant differences between the groups

	LI-H	LI-G	LII-H	LII-G	LIII-G
LI-H	▧	10	15	20	14
LI-G	▧	▧	2	10	9
LII-H	▧	▧	▧	9	6
LII-G	▧	▧	▧	▧	1

## Discussion

To our knowledge, this is the first study to examine the influence of transport mode and hospital level on mortality in a certified German trauma network (TraumaNetzwerk DGU^®^).

The main findings are that there were no differences in unadjusted and adjusted mortality by hospital level, mode of transport, or both in our results, but there were clear differences between the study groups in patient characteristics.

The literature does not provide clear evidence of a survival advantage for trauma patients transported by HEMS compared to GEMS. A literature review from 2005 showed a survival advantage for HEMS transports [38]. Tsuchiya et al. found the same for a large Japanese cohort in 2016 [25]. A Cochrane analysis from 2015 found no significant difference [39]. Beaumont et al. observed a trend toward improved survival rates with HEMS transport in an English cohort in 2020 [40]. German authors consistently found an advantage for HEMS transports [22–24, 41]. However, the international publications did not include trauma networks. Additionally, German publications were based on data collected before Germany was fully covered by trauma networks in 2015. Two publications correlate the survival rate not only with the type of transport but also with the level of the destination hospital. In 2004, Biewener et al. showed a survival advantage for the region around Dresden through the combination of HEMS and treatment in a university hospital [22]. Using the TraumaRegister DGU^®^ database with cases from 2005 to 2011, Schweikofler et al. demonstrated a survival benefit when combining HEMS transportation and treatment in a Level I hospital [41]. All German publications mentioned, with the exception of Schweikofler’s showed a rather homogeneous distribution of patient characteristics between HEMS and GEMS [22–24]. This was not the case in our evaluation. Patient characteristics were significantly different for age, ASA, injury severity and mortality risk. The most seriously injured patients were transferred to Level I hospitals. The helicopter was used to transport more seriously injured and younger patients to the Level I and II hospitals than GEMS. However, GEMS transports to Level I and II hospitals revealed a higher percentage of TBI than HEMS transports. Combined with the older age of the GEMS patients and the higher ASA values, this could be an expression of the age-related trauma patients with TBI [42]. In this regard, it should be emphasized that the expected mortality (RISC II) was highest in the LI-G group, not in the LI-H group. But the oldest patients with the least severe injuries were admitted to Level III hospitals. They also had the lowest percentage of severe traumatic brain injuries.

This result could only be achieved through adequate preclinical patient assessments, which served as the basis for transportation and destination hospital decisions. Germany’s emergency physician system could facilitate this. It includes ground-based emergency physicians who are alerted to emergencies in accordance with the reporting pattern, particularly in cases of severe trauma. An improved survival rate was also found for an English cohort with additional physician care for GEMS transport [40]. Providing the preclinical team with good information about the service options of the respective hospitals within a certified trauma network improves the decision-making process for selecting the right destination hospital. McQueen et al. showed clear positive effects of the introduction of a trauma network in the UK on air ambulance operations and reduced the number of false alarms [12]. The implementation of trauma network structures has been identified as having a positive impact on survival rates of patients, regardless of type of transportation [21, 43].

Overall, the results of our study and the existing literature indicate that combining the trauma network philosophy with appropriate transportation to the most relevant hospital, based on an accurate on-site assessment, is key to success.

It should be noted that we found a significant time advantage for GEMS-transported patients for both Level I and Level II hospitals. This is partly due to the high hospital density in Germany [21, 44]. In countries with a high hospitalization rate, ground transport is often faster than HEMS [45]. Particularly at night, a lead time of up to 30 min must be expected before the helicopter takes off due to safety measures [46]. Accordingly, rescue at night in the study region is two-staged: the ground-based rescue service is always primarily on site, with HEMS alerted if necessary. Even during the day, ground transportation has a time advantage for distances of up to 30–50 km [45].

The significantly higher intubation rate of HEMS patients may also have contributed to the longer duration of prehospital care in this study. Preclinical intubation is more likely to be indicated for trauma patients in the case of HEMS transport, as it is very difficult to perform intubation during flight due to the confined space of the helicopter. However, despite all the attention paid to the Golden Hour of Shock, it has been shown for trauma patients with blunt injuries that the preclinical time has little influence on the survival rate [47, 48].

In terms of process quality, it should be emphasized that there was no significant difference between the care levels in the primary performance of CT diagnostics. The gold standard for trauma diagnostics [49], was available and used for all patients, regardless of the level of the hospital. However, there were significant differences in the time to CT, with the maximum in the Level III clinics (45.4 min.) and the minimum in the LII-H group (18.3 min.). During the observation period, there was no clinic with integrated CT in the trauma room and no clinic with CT first protocol [50]. In order to be certified as a trauma center, the implementation of an in-house trauma room algorithm is mandatory [14]. Nevertheless, the maximum time spent in Level III hospitals suggests that improvements could still be made to trauma room care processes at this level. In terms of the clinical course, Level III hospitals had the second-highest percentage of intensive care patients (93.0%), which was higher than that of both groups of Level II hospitals (LII-H: 87.0%; LII-G: 89.6%) and the Level I-G group (92.1%). It should be noted that patients who died in the trauma rooms of Level I and II hospitals play a role in the distribution. If these patients were hypothetically included in the ICU admissions, the percentage of 93% for the LIII-G group would be lower than that of the LI-G and LII-G groups. Nevertheless, Level III hospitals had the lowest injury severity and intubation rates. Even though these differences were not significant in the post-hoc analysis, and the number of patients examined in Level III hospitals was relatively small, the question arises as to whether the high rate of intensive care admissions could be reduced for economic reasons.

While the length of stay in the intensive care unit and the length of intubation showed significant differences between the study groups, the total length of hospital stay was comparable with a trend for a longer stay in the LI-H group. These mirror the analogous findings for unadjusted and adjusted mortality outcomes. The clinics’ comparable outcomes were based on selection in the preclinical phase, as reflected by differences in patient characteristics. The number of significant differences between the study groups in the post-hoc analysis could be another indication of their distinction. The LI-H group showed 10 or more significant differences compared to all other groups in this evaluation, while LI-G/LII-H and LII-G/LIII-G showed less than 3 significant differences.

In the evaluated trauma network, the 40-year-old D. Trunkey requirement to bring the right patient to the right hospital at the right time (the 3-R rule [51]) could be fulfilled and expanded to include the parameter of the right type of transportation.

### Limitations

The study has several limitations:

1. Retrospective analysis

The research was a multicenter study of all the trauma centers in a trauma network in a predominantly rural area. Despite the prospective data collection in the individual clinics and the focus on the highest possible data quality [21, 27], the present study is retrospective due to the secondary research question on the data set. We do not assume that this has resulted in a bias, as the prospectively collected data was not changed.

2) Register research

The data set of the TraumaRegister DGU^®^ served as the data basis, a registry research data set. The data set is internationally recognized, verified and Utstein-compliant [3, 28, 29]. Nevertheless, the dataset is a compromise to provide variables for different questions. Further extended questions in depth cannot be answered with the available data set. However, the three dimensions of data quality—completeness of patients, completeness of recorded variables, and accuracy—were met to a very high degree due to the study’s setting. In a health services research study like ours it seems reasonable to use a certified register.

3) Non-recording of out-of-hospital deaths

Patients who did not reach the hospitals and died prehospital could not be included due to the study design. Since the primary endpoint of the study was hospital mortality as a function of treatment received at the hospital and the type of transport, including prehospital deaths would not change the results.

4) Transferability of the results

The study comes from a high-income country with a dense network of hospitals, an emergency physician system even for GEMS and a low proportion of penetrating injuries. Accordingly, the results of this study cannot be transferred to other parts of the world without further ado. However, we assume that a positive influence on mortality through the formation of trauma networks would be recognized all over the world. In countries with significantly longer transport distances, than those in our study, HEMS transport could be advantageous. For a patient population with more penetrating injuries, the fastest mode of transport would likely offer a survival advantage.

To overcome these limitations and to confirm our study results, it seems reasonable and necessary to conduct further studies in different trauma networks in different countries.

## Conclusion

The mortality of severely injured patients in our trauma network was not affected by the hospital level or mode of transport (GEMS/HEMS). This was because the patients were thoroughly evaluated in the prehospital setting and transported to the appropriate hospitals by the most suitable means. Further simplification and improvement of patient selection would be useful [52]. Modern vehicles measure and store the forces that act in the event of an accident (event data recorders, EDRs) [53]. Unfortunately, these measurements have not yet been handed over to the emergency services. In our opinion, this would have the potential to provide accident victims with the necessary treatment more quickly and efficiently.

## Data Availability

The dataset generated and analyzed during the current study is not publicly accessible but is available from the corresponding author on reasonable request.
